# LOTUS overexpression accelerates neuronal plasticity after focal brain ischemia in mice

**DOI:** 10.1371/journal.pone.0184258

**Published:** 2017-09-07

**Authors:** Hajime Takase, Yuji Kurihara, Taka-akira Yokoyama, Nobutaka Kawahara, Kohtaro Takei

**Affiliations:** 1 Department of Neurosurgery, Yokohama City University Graduate School of Medicine, Yokohama, Japan; 2 Molecular Medical Bioscience Laboratory, Department of Medical Life Science, Yokohama City University Graduate School of Medical Life Science, Yokohama, Japan; Universita degli Studi di Napoli Federico II, ITALY

## Abstract

Nogo receptor-1 (NgR1) and its ligands inhibit neuronal plasticity and limit functional recovery after brain damage such as ischemic stroke. We have previously shown that lateral olfactory tract usher substance (LOTUS) antagonizes NgR1-mediated signaling. Here, we investigated whether LOTUS enhances neuronal plasticity and functional recovery after brain focal ischemia in adult mice. Focal ischemic infarcts were induced in wild-type and LOTUS-overexpressing transgenic mice via middle cerebral artery occlusion. Endogenous LOTUS expression was increased in brain and cervical spinal cord of the contralateral side of ischemia in the chronic phase after brain ischemia. LOTUS overexpression accelerated midline-crossing axonal sprouting from the contralateral side to the ipsilateral side of ischemia in the medullar reticular formation and gray matter of denervated cervical spinal cord. Importantly, LOTUS overexpression improved neurological score highly correlated with laterality ratio of corticoreticular fibers of the medulla oblongata, indicating that LOTUS overexpression may overcome the inhibitory environment induced by NgR1 signaling for damaged motor pathway reconstruction after ischemic stroke. Thus, our data suggest that LOTUS overexpression accelerates neuronal plasticity in the brainstem and cervical spinal cord after stroke and LOTUS administration is useful for future therapeutic strategies.

## Introduction

Stroke is one of the most important human health concerns worldwide, leading to cognitive and motor function decline that requires long-term social support. Although there are several injurious cascades in the acute phase after stroke and some compensatory reactions in the chronic phase, the mechanisms underlying neuronal plasticity and critical therapeutic targets for functional recovery after stroke remain unclear.

Motor deficits after stroke are primarily caused by damage to cortical motor neurons or by interruption of excitatory transduction in the long axons that innervate (directly or indirectly) secondary motor neurons in spinal cord gray matter [1–4]. Numerous studies have been conducted to overcome the damage and interruption of excitatory transduction in motor-related pathways in the central nervous systems (CNS) [5–8].

Axon growth inhibition by myelin-associated inhibitors is a crucial obstacle of functional recovery in the damaged adult CNS [9]. Axon growth inhibitors, such as Nogo proteins, bind to the Nogo receptor-1 (NgR1), which is expressed in many types of neurons in CNS. Axon growth inhibitor binding to NgR1 leads to the limitation of neuronal plasticity and functional recovery in animal models of ischemic stroke in the chronic phase [10, 11]. To overcome NgR1-mediated axon growth inhibition, several approaches for blocking the binding of these ligands to NgR1 have been performed in ischemic stroke [5, 7, 10–13]. However, the effects of these approaches are limited by compensation by multiple axon growth inhibitors [14].

Lateral olfactory tract usher substance (LOTUS) shows an almost complete blockade against NgR1-mediated axon growth inhibition by its ligands *in vitro* [15, 16]. Moreover, LOTUS is an endogenous protein and administration using an endogenous protein is logical and advantageous for future therapy. We hypothesized that LOTUS may counteract NgR1-mediated axonal growth inhibition *in vivo*, thereby promoting neuronal regeneration as a potent NgR1 inhibitor after stroke. It is thus effective as prolonged treatment through the chronic phase by minimizing the compensatory effects of multiple axon growth inhibitors.

In the present study, we examined physiological changes in LOTUS expression under ischemic conditions and used LOTUS overexpressing transgenic mice to assess the role of LOTUS in histopathological conditions of motor-related pathways after experimental stroke.

## Materials and methods

### HA-LOTUS transgene construction and generation of transgenic mice

LOTUS-overexpressing transgenic mice (LOTUS-Tg) were generated with the use of the synapsin-1 promoter, which directs neuron-specific LOTUS expression. The LOTUS transgene was constructed by insertion of the HA-fused mouse lotus gene (85–1938 base) into the Igk chain secretion signal and rabbit β-globin intron/polyA. The purified plasmid was then microinjected into the pronuclei of fertilized mouse oocytes derived from C57BL/6J mice (CLEA Japan, Inc., Tokyo, Japan) (S1A Fig). Microinjected oocytes were then implanted into pseudopregnant females of the same mouse strain. After screening DNA samples from the litters, we bred transgenic founders with wild-type (WT) mice to establish transgenic lines. LOTUS overexpression was confirmed in the CNS of WT mice, and in heterozygous and homozygous LOTUS-Tg mice (S1B and S1C Fig).

### Animals

Forty-one WT and thirty-nine homozygous LOTUS-Tg male mice 12 to 14-weeks-old and (C57BL/6J; CLEA Japan, Inc., Tokyo, Japan) weighing 28–33 g, which was chosen to obtain lower mortality in this MCAO model, were used. The mice were housed in a standard mouse facility with free access to autoclaved diet and water. This study was carried out in strict accordance with the recommendations in the Guide for the Care and Use of Laboratory Animals of the National Institutes of Health. All procedures in this study were approved by the institutional Animal Care and Use Committee of the Yokohama City University (Permission Number: F-A-15-047). All surgery was performed under isoflurane anesthesia, and all efforts were made to minimize the number of animals used and their suffering throughout the experimental procedures.

Thirty-two out of 41 WT and 30 out of 39 LOTUS-Tg mice were subjected to middle cerebral artery occlusion (MCAO). Six mice from each group were used for a sham operation, and the other 3 mice from each group were used as preischemic controls in immunoblot analysis. To assess the infarct volume 72 h after MCAO, 9 out of 32 WT and 6 out of 30 LOTUS-Tg mice subjected to MCAO were used (S2A Fig). Nine out of 32 WT and 30 LOTUS-Tg MCAO mice, and 6 sham of both WT and transgenic animals were assessed at each time point for behavioral performance, then sacrificed for histological examination (S2B Fig). For immunoblot analysis, 3 preischemic normal mice in both groups were used as controls and 3 MCAO mice 6 and 16 weeks after stroke were used as ischemic animals, respectively (n = 9 in each group, n = 3 at each time point) (S2C Fig).

After MCAO, the body weight of all animals was measured three times in the first week, and the mice weighing from −8 to −3 g in comparison with preoperative weight were used for the following analysis to confirm the homogenous severity of insults [17, 18]. In addition to the body weight loss, we also confirmed success of MCAO surgery by using the Bederson’s neurological score [19, 20]. There was no significant difference in body weight between WT and LOTUS-Tg mice (S4 Fig). Five out of 32 WT and 6 out of 30 LOTUS-Tg MCAO mice were excluded by this body weight criteria. Of those who were excluded, 1 from each group was excluded on the basis of the lack of body weight loss after MCAO. Two from both groups were excluded by neurological score. Three from both groups were dead 3 days after MCAO.

### Middle cerebral artery occlusion

To induce focal cerebral ischemia, mice were anesthetized with 1.5% isoflurane (Pfizer Japan Inc., Tokyo, Japan) in room air using a facemask. Rectal temperature was maintained between 36.5 and 37.0°C using an electrical heating pad (Bio Research Center Co. Ltd, Nagoya, Japan; Physitemp Instruments Inc., Clifton, NJ). Focal cerebral ischemia was induced using an intraluminal filament procedure [21, 22]. Briefly, a midline skin incision of the neck was made, and right common and external carotid arteries were isolated and ligated. A microvascular clip temporarily clipped the right internal carotid artery. A 50 mm 7–0 surgical monofilament nylon suture (custom made), coated at the tip with silicone, was introduced through a small incision into the right common carotid artery and advanced 9 to 10 mm distal to carotid bifurcation for MCAO. The surgical procedure of MCAO did not exceed 10 minutes. During MCAO, mice were once awakened after skin closure, and then anesthetized again after 40 min of MCAO. Blood flow was restored by withdrawal of the nylon suture after 45 min of MCAO. The right internal carotid artery was exposed and ligated. During anesthesia, Laser Doppler Flowmetry (LDF) (Advance Co., Ltd., Tokyo, Japan) was used to check appropriate reduction of cerebral blood flow (CBF) in MCA area by 85% of preocclusion value in the first 5 min of MCAO, and to confirm reperfusion after removal of the suture (S3C Fig).

### Stroke outcome/histological assessment of brain damage following MCAO

Animals were sacrificed via decapitation 72 h after MCAO and brain tissues were collected immediately. The brain tissues were cut into 7 serial coronal sections of 1-mm thickness, then stained with 2% 2,3,5-Triphenyl-tetrazolium chloride (TTC, Sigma–Aldrich, Germany) in 0.01 M phosphate-buffered saline (PBS) for 20 min at 37°C in a dark room. The images of the stained sections were captured by a digital camera (Canon, Tokyo, Japan) and analyzed using ImageJ software (NIH). The area of intact tissue in the ipsi-ischemic hemisphere was subtracted from the area of the contra-ischemic hemisphere (% Infarct Area = ((A_contra_ −A_ipsi_)/A_contra_) × 100) [23, 24] (S2A Fig).

After perfusion fixation in 19 weeks after MCAO, the brains were removed, seven 20 μm coronal sections spaced at 1 mm intervals throughout the forebrain of each animal 19 weeks after MCAO were stained with cresyl violet acetate (Muto Pure Chemicals Co., Ltd., Tokyo, Japan) for assessment of the ischemic damage of the brain hemisphere in the chronic phase (S2B Fig). Images of each section were digitized and the infarct area was measured with ImageJ software. To evaluate the damage of the total hemisphere 19 weeks after MCAO, we also examined the degree of cortical cavitation using a “cortical width index” as described previously [25]. The cortical width index was calculated by dividing the edge-to-midline distance on the ischemic side by that on the non-ischemic side [26].

### Immunoblot analysis of LOTUS, NgR1, and Nogo-A

At the time before and 6 and 16 weeks after stroke (n = 3 at each time point in both groups), tissue samples were selectively dissected after cardiac perfusion with PBS and divided into non-ischemic and ischemic side of the hemisphere from basal ganglia to the cervical spinal cord at 4°C. Those samples were then homogenized in immunoprecipitation buffer [20 mM Tris-HCl, pH 8.0, 150 mM NaCl, 1 mM EDTA, 10 mM NaF, 1 mM Na3VO4, 1% Nonidet P-40, 50μM ρ-amidinophenylmethanesulfonyl fluoride, and 10 μg/mL of aprotinin] using a hand-held homogenizer. The lysates were centrifuged at 1500 rpm for 10 min at 4°C. Next, 20 μg of soluble proteins were separated by 8% SDS-polyacrylamide gel electrophoresis and subsequently electrotransferred for 1 h at 300 mA at room temperature (RT) onto polyvinylidene difluoride membranes (Immobilon-P, Millipore, Bedford, MA). The membranes were blocked for 1 h with 5% skim milk in Tris-buffered saline with 0.1% Tween 20 (TBST) and incubated with primary antibodies for 24 h at 4°C followed by incubation with secondary antibodies for 1 h at RT. Proteins were detected by using polyclonal guinea-pig anti-LOTUS antibody (1:2000 dilution; Medical & Biological Laboratories Co., Ltd., Nagoya, Japan), polyclonal goat anti-NgR1 (1:2000 dilution; R&D Systems, Inc., Minneapolis, MN), monoclonal mouse anti-Nogo A (1:500 dilution; Merck Millipore, Billerica, MA) and monoclonal mouse anti-β actin (1:20000 dilution, Sigma-Aldrich, St. Louis, MO) [27]. Horseradish peroxidase-conjugated goat anti-guinea pig antibody (1:2000 dilution; Jackson ImmunoResearch Laboratories, Inc., West Grove, PA), donkey anti-goat (1:2000 dilution; Jackson ImmunoResearch Laboratories, Inc., West Grove, PA), donkey anti-mouse IgM (1:2000 dilution; Jackson ImmunoResearch Laboratories, Inc., West Grove, PA) and sheep anti-mouse IgG (1:2000 dilution; GE Healthcare Bio-Sciences Corp., Piscataway, NJ) were used as secondary antibodies. All antibodies were diluted in 1% skim milk in TBST. Immunoblots were visualized using enhanced chemiluminescence (ECL) according to the manufacturer’s specifications. For quantitative analysis, ECL images (ImageQuant 400; GE Healthcare Life Sciences, Piscataway, NJ) were analyzed using ImageJ software.

### Anterograde tracing of motor pathways

Seventeen weeks after MCAO and/or sham operation, animals were secured in a stereotactic frame, and a unilateral craniotomy was performed with a high-speed drill over the left frontal motor cortex, leaving the dura mater completely intact. Anterograde tracer, biotinylated dextran amine (BDA, 10,000 MW; Molecular Probes, Eugene, OR), was injected into four locations in the motor cortex contralateral to the ischemic stroke site as previously described, with slight modification [28, 29]. Briefly, a 10% solution of BDA in 0.1M phosphate buffer (pH 7.4) was injected through a finely drawn glass capillary (200 nL per injection site; stereotaxic coordinates: 0 and 0.5 mm rostral to the bregma, 1.5 and 2.0 mm lateral to the midline, 0.7 mm deep from the cortical surface) to label the corticospinal tract (CST) axons originating from the pyramidal neurons. Two weeks after the tracer injection, mice were transcardially perfused with PBS containing heparin followed by 4% paraformaldehyde (PFA) in PBS. Brains and cervical spinal cords were dissected and post-fixed overnight in 4% PFA in PBS containing 5% sucrose, and thereafter cryoprotected in increasing concentrations of sucrose (10% and 30%) over 3 days.

Tissues were then frozen and cut into coronal sections of 20 and 50 μm thickness for brains for cresyl violet and BDA staining, respectively; and 50μm in spinal cords for BDA staining.

For the detection of BDA, sections were incubated for 2 h with a Vectastain ®ABC kit (Vector Laboratories, Burlingame, CA, USA). Staining deposits were visualized with 3,3'- diaminobenzidine tetrahydrochloride (Molecular Probes, Eugene, OR) [30]. For evaluation of BDA-labeled axonal projections in brain, axonal projections from the cerebral cortex to the reticular formation (RF) were evaluated at the level of the medulla oblongata (−5.5 to −6.0 mm caudal to bregma). Two parallel, 1 mm-long lines from the lateral and medial (midline) edges of the corticospinal tract (CST) were superimposed on the sections. Along those lines, fibers from CST, without and with crossing midline, to contra- and ipsi-ischemic RF were quantified, respectively. The total number of those fibers was normalized with the total number of BDA-labeled fibers in the CST as previously described [31]. Two consecutive sections were analyzed and mean values were determined. Laterality index was also evaluated by the ratio of fibers (those in non-ischemic side/those in ischemic side). In spinal cord sections, the cervical spinal cord segments at C4-5 and C6-7 levels were respectively analyzed to examine which was significantly restored with the forelimb flexor muscle, Biceps Brachii (mainly innervated by ventral horn cells of C4-5) or extensor, Triceps Brachii (C6-7) [4]. The total number of midline-crossing axons in the denervated (non-ischemic) side of the ventral gray matter was counted. All fiber counting were assessed in a blind manner. Sections were evaluated and analyzed with ImageJ software (NIH, version 1.48).

### Neurological score

In animals subjected to behavioral analysis, neurological status of the forelimb and upper body was evaluated according to the score described by Bederson et al with slight modification [18, 32, 33]. The scoring was performed just before MCAO, and 1, 4, 12, and 16 weeks after MCAO. Briefly, each mouse was held gently by the tail, and observed for body swing and forelimb flexion of the contra-lesion side, which suggested gross motor disability of the upper body trunk and forelimb, respectively. Mice that extended both forelimbs toward the floor were then allowed to move freely and were observed for leftward circling behavior [18, 32–34]. The examination was performed for 3 min. Animals that had no neurological deficit were assigned a score of 0, while those that had 1 and 2 out of those three deficits were assigned a score of 1 and 2, respectively, and all three deficits were assigned a score of 3. All experiments were performed and quantified in a randomized fashion by investigators blinded to mouse genetic background.

### Statistical analysis

Data were expressed as mean ± S.E.M. and analyzed by unpaired *t* test (comparisons of infarct volume, cortical width index and laterality index), two-way ANOVA with Tukey post hoc analysis (comparisons between more than three groups), or repeated-measures ANOVA (comparisons at more than 2 time-points), as appropriate. These calculations were performed using Prism software (GraphPad Software, Inc., La Jolla, CA). Differences were considered significant at *p* < 0.05.

## Results

### LOTUS overexpression does not influence brain atrophy or cortical cavitation after ischemic stroke

**Fig 1:**
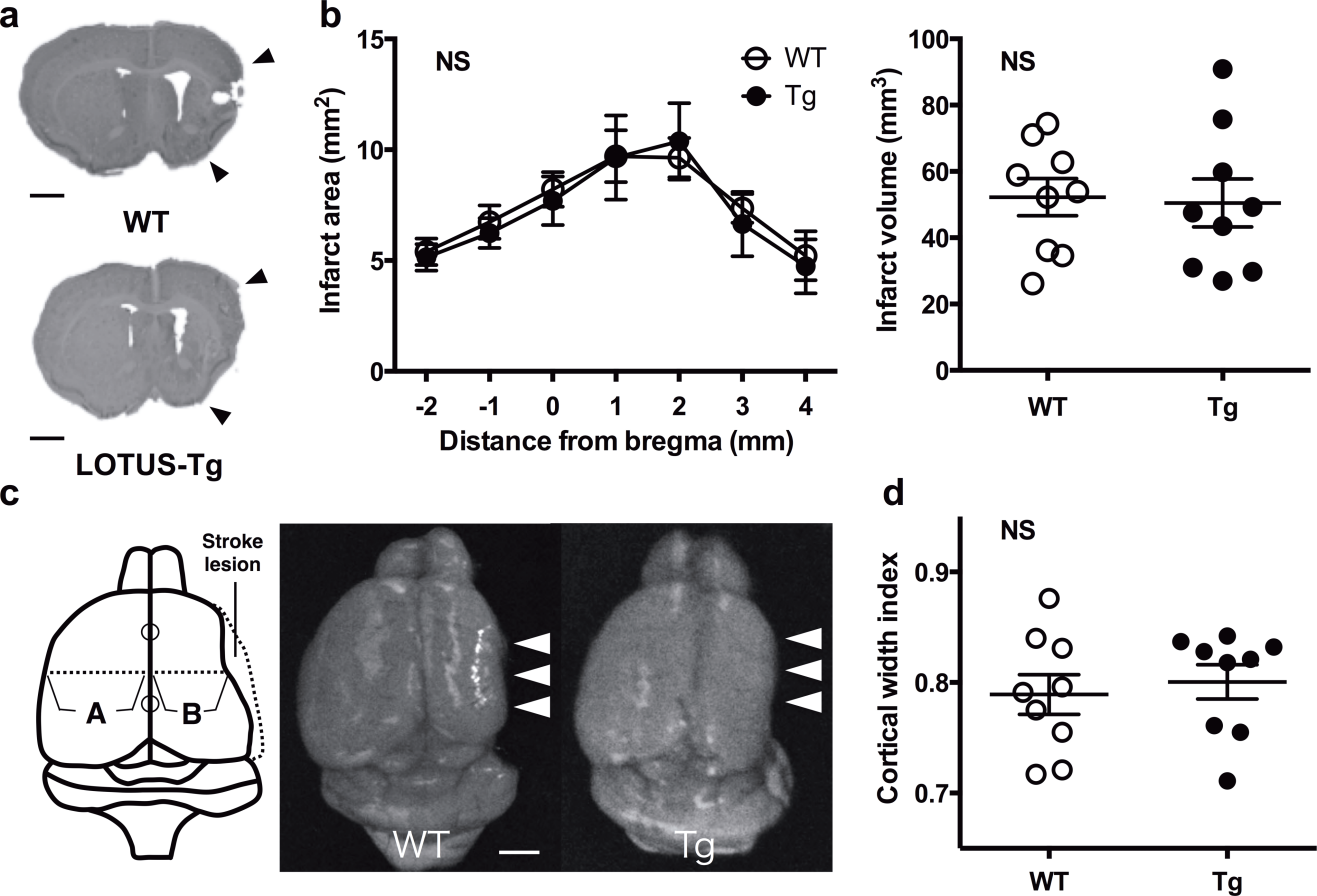
No influence of LOTUS overexpression in brain atrophy and cortical cavitation in the chronic phase after ischemic stroke. (**a**) Representative coronal sections stained with cresyl violet of brain in WT and LOTUS-Tg mice 19 weeks after MCAO. Arrowheads indicate edge of stroke lesion. (**b**) Infarct area (mm^2^) in each slice and volume (mm^3^) in WT and LOTUS-Tg mice. (Infarct area: 2-way ANOVA; NS. Data are mean ± S.E.M. n = 9 per group, Infarct volume: Unpaired *t* test; NS. Data are mean ± S.E.M. n = 9 per group) (**c**) Left, cortical width index; maximum width from midpoint to edge of non-infarcted (A) and infarcted (B) hemispheres. Right, dorsal view of WT and LOTUS-Tg mouse brains. White arrowheads indicate infarct region. (**d**) Quantitative analysis of cortical width index. (Unpaired *t* test; NS. Data are mean ± S.E.M. n = 9 per group) Bar indicates 1 mm. NS, not significant.

First, we examined whether LOTUS overexpression in LOTUS-Tg mice influenced cerebral infarction both in the acute and chronic phase after MCAO. Ischemic regions in the acute phase and brain atrophy in the chronic phase were clearly observed in the cortex and striatum of WT and LOTUS-Tg mice subjected to 45 min MCAO (Fig 1A, S3A Fig). No statistical difference was found in infarct area and volume with similar CBF during MCAO, in addition to the body weight between WT mice and LOTUS-Tg mice (Fig 1B, S3B and S3C Fig, S4 Fig, S1 Table). In cortical width index, cortical cavitation showed no significant difference between WT and LOTUS-Tg mice (Fig 1C and 1D, S1 Table). These data indicate that LOTUS overexpression in LOTUS-Tg mice does not induce cerebral cortical expansion both after acute and chronic ischemic stroke compared with that in WT mice. Therefore, we compared the spatiotemporal protein expression such as NgR1, Nogo-A and LOTUS and the histopathological condition of axonal remodeling in the motor pathway between WT and LOTUS-Tg mice after MCAO. Moreover, we also investigated the ischemia-induced global neurological deficits and the correlation between their neurological improvements and pathological conditions to examine whether axonal remodeling was functional in those ischemic mice.

### Ischemic stroke increases LOTUS expression in non-ischemic regions in the late stage after MCAO

**Fig 2:**
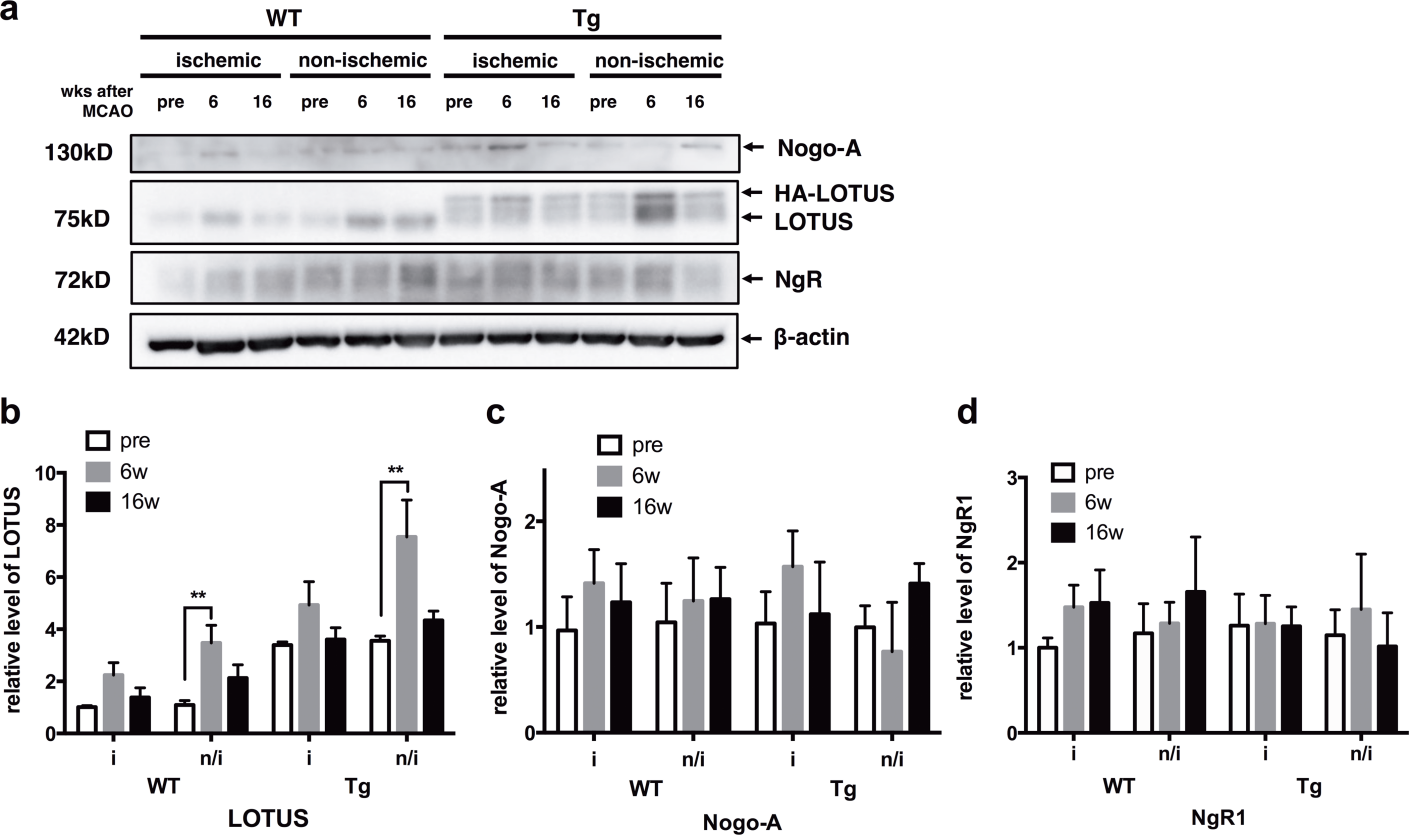
LOTUS, Nogo-A and NgR1 expression in WT and LOTUS-Tg mice. (**a**) Protein (20 μg) from lysates of each animal in pre-stroke, 6, and 16 weeks after MCAO was analyzed. (**b**-**d**) Quantitative analysis of expression level in LOTUS, Nogo-A, and NgR1. Immunoblots were normalized with β-actin. (**b**) Increases in LOTUS expression in the non-ischemic side of both WT and LOTUS-Tg mice 6 weeks after MCAO were found. (**c**, **d**) No significant difference was seen in Nogo-A and NgR1 expression. (2-way ANOVA with Tukey post hoc analysis; **p < 0.01. Data are mean ± S.E.M. n = 3).

Previous reports suggested that ischemic stress would elicit spatiotemporal changes in CNS protein expression including NgR1-related ligands [35, 36]. Thus, we next examined expression pattern of LOTUS, its binding partner NgR1, and Nogo-A, a ligand of NgR1 in the ischemic region and non-ischemic region contralateral to the ischemic side after MCAO. Immunoblots revealed that protein expression levels of LOTUS in the CNS tissue of LOTUS-Tg mice, including basal ganglia, brain stem, and cervical spinal cord, were higher than those in WT mice, as expected. Significant increases in LOTUS expression were observed in non-ischemic sides at 6 weeks after MCAO in both WT and LOTUS-Tg mice, but not in the ischemic side in WT or LOTUS-Tg mice (Fig 2A and 2B). In contrast, the expression levels of NgR1 and Nogo-A were unchanged at all time points in both WT and LOTUS-Tg mice (Fig 2C and 2D). These findings suggest that ischemic stroke induces an increase in endogenous LOTUS expression in WT mice and overexpressed LOTUS in LOTUS-Tg mice in the non-ischemic region contralateral to the ischemic side in the late stage after MCAO. The increase of LOTUS expression in the non-ischemic region may be a compensatory response towards ischemic stroke-induced neuronal plasticity inhibition.

### LOTUS enhances neuronal plasticity of motor pathways

Because ischemic stroke increased LOTUS expression in the non-ischemic region, we histologically examined whether neuronal plasticity is also enhanced in the non-ischemic region after MCAO. CST was labeled with BDA injected into the non-ischemic side of the motor cortex and labeled CST axon fibers sprouting from descending fibers in the non-ischemic side of the medullary RF and cervical spinal cord were examined.

In all animals, the BDA injection areas in the non-ischemic side covered the caudal forelimb area of the primary motor cortex without spreading tracer deposits into subcortical structures. No difference was observed in BDA tracer injection areas of WT and LOTUS-Tg mice. We first examined whether LOTUS overexpression in LOTUS-Tg mice influenced the formation of CST fibers. The number of BDA-labeled CST fibers at the level of the medullary RF in LOTUS-Tg mice was not different from that in WT mice (S2 Table).

**Fig 3:**
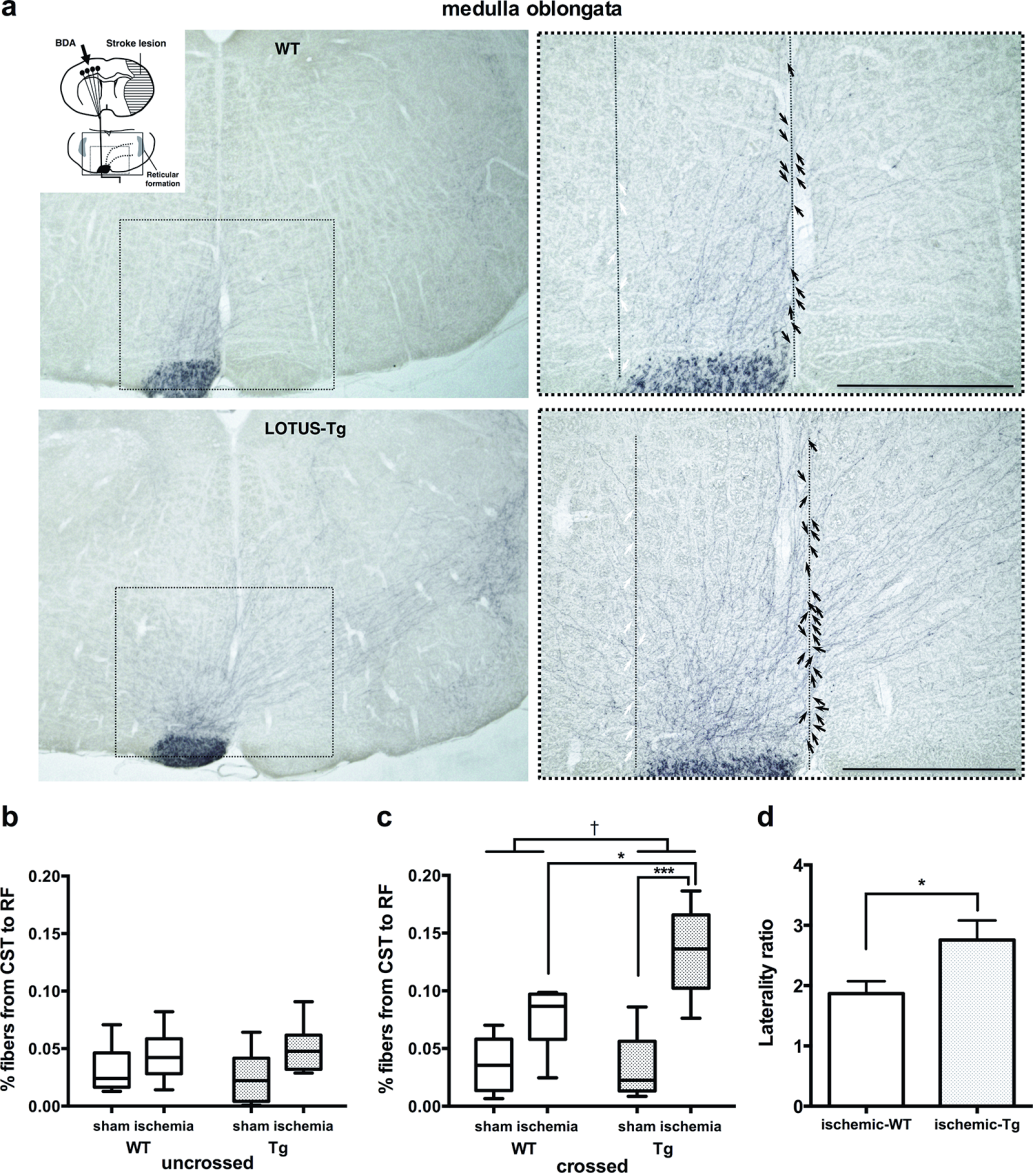
Neuronal remodeling in cortico-reticular fibers. (**a**) Representative photomicrographs of the medulla oblongata in frontal sections of WT and LOTUS-Tg mice, showing increased midline (dashed lines)-crossing corticoreticular fibers (black arrows) from non-ischemic CST in LOTUS-Tg mice after MCAO without influencing uncrossed corticoreticular tact (white arrows on dashed lines). Insets of the scheme indicating the position of the photomicrograph. Images in right panel are shown at higher magnification of indicated dashed boxes. Scale bars indicate 500 μm. (**b, c**) Percentage of uncrossed and midline-crossing fibers from CST to RF (2-way ANOVA with Tukey post hoc analysis; *p < 0.05, ***p < 0.001, ^†^*p* < 0.05. Data are interquartile range. n = 9 per group). (**d**) Laterality ratio comparing WT and LOTUS-Tg mice. (Unpaired *t* test, *p < 0.05. Data are mean ± S.E.M. n = 9).

We then quantified the number of BDA-labeled CST fibers crossing the midline towards the ischemic side in RF, as well as those uncrossing in the non-ischemic side (Fig 3A). In the crossing fibers, statistical analysis by 2-way ANOVA revealed that BDA-labeled CST fibers were significantly increased by ischemia and LOTUS overexpression (*p* < 0.001, *p* = 0.040, respectively; interaction *p* = 0.024, *F* = 8.72). By the post hoc test, the numbers of these fibers in LOTUS-Tg mice were significantly higher than those in WT mice after MCAO (Fig 3C, S3 Table). In the uncrossing fibers, however, no significant difference was found (Fig 3B, S3 Table). These data show that the increase in crossing fibers was likely induced by LOTUS overexpression related to ischemic stress. The laterality ratio (%) calculated as the number of crossing fibers divided by that of uncrossing fibers in LOTUS-Tg after MCAO was also statistically higher than those in WT after MCAO (Fig 3D, S3 Table).

**Fig 4:**
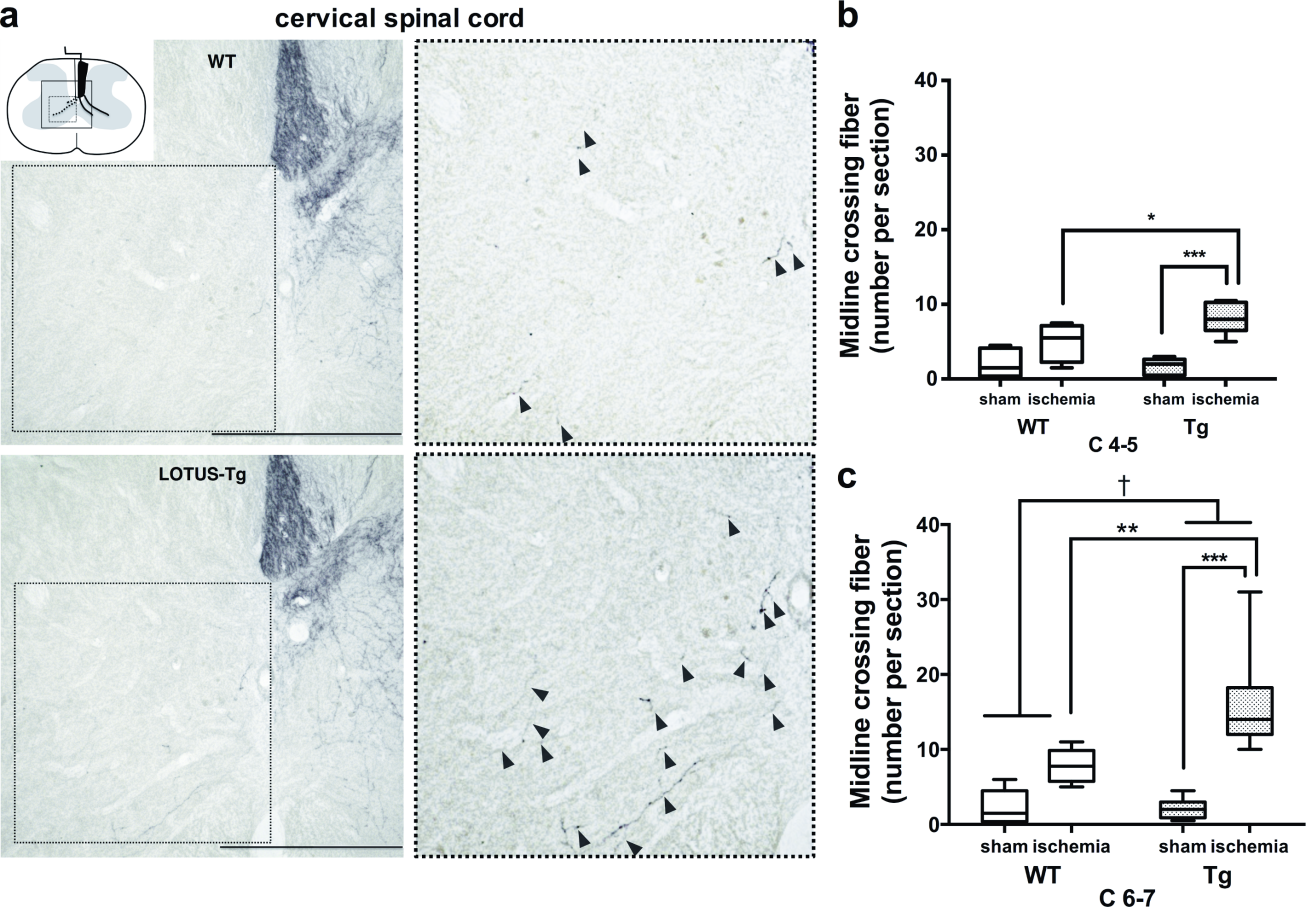
Neuronal remodeling in cortico-spinal fibers. (**a**) Representative photomicrographs of the gray matter of the cervical spinal cord in frontal sections of WT mouse and LOTUS-Tg mouse, showing increased midline-crossing fibers (black arrowheads) in LOTUS-Tg mice after MCAO. Insets of the spinal cord scheme indicating the position of the photomicrograph. Images in right panel are shown at higher magnification of indicated dashed boxes. Scale bars indicate 500 μm. (**b, c**) The number of midline-crossing fibers of WT and LOTUS-Tg mice at the C4-5 and C6-7 levels of the cervical spinal cord. (2-way ANOVA with Tukey post hoc analysis; **p < 0.01, ***p < 0.001, ^†^*p* < 0.05. Data are interquartile range. n = 9 per group).

Next, the number of CST midline-crossing fibers in the denervated (contra-ischemic) side was counted in the gray matter of the cervical spinal cord at the C4-5 and C6-7 levels (Fig 4A). At the C6-7 levels, both ischemia and LOTUS overexpression significantly increased these fibers (2-way ANOVA, *p* < 0.001, *p* = 0.020, respectively; interaction *p* = 0.016, *F* = 6.86). The post hoc analysis revealed that the numbers of the fibers in LOTUS-Tg mice were significantly higher than those in WT mice after MCAO. In LOTUS-Tg mice, the numbers of the fibers after MCAO were also higher than those without MCAO (Fig 4C, S4 Table). Similar results were obtained at the C4-5 levels (Fig 4B, S4 Table), although no statistical significance was seen between WT and LOTUS-Tg mice (*p* = 0.056). Thus, the data show that the midline-crossing CST fibers at the cervical spinal cord increased in LOTUS-Tg mice after MCAO, particularly at the C6-7 level.

These findings suggest that upregulated endogenous and overexpressed LOTUS expression caused by ischemia may increase crossing fibers of the cortico-reticular tract (CRT) and CST, leading to enhancement of neuronal plasticity after MCAO.

### LOTUS overexpression promotes functional recovery after ischemic stroke

**Fig 5:**
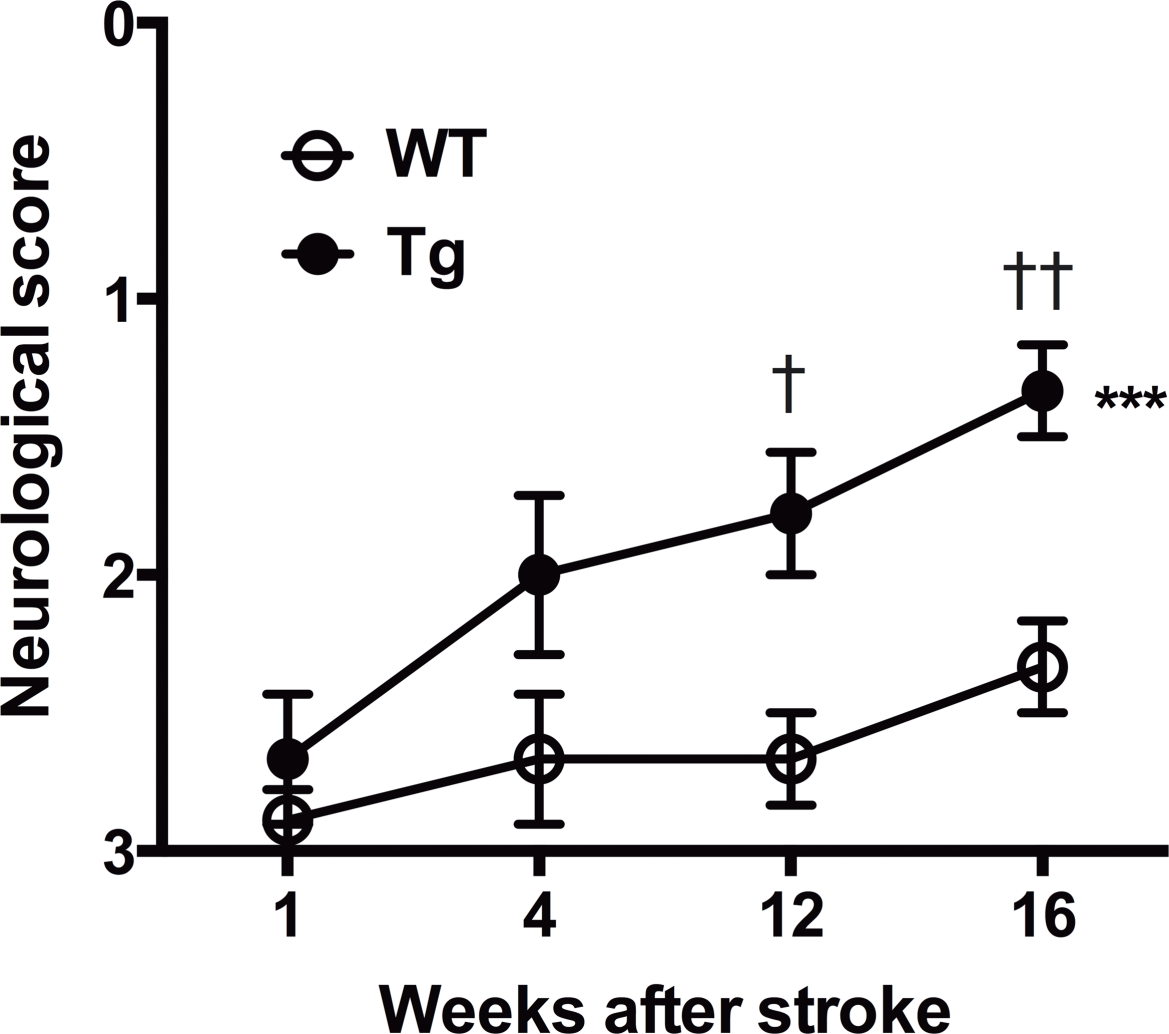
Behavioral outcome after MCAO. Behavioral outcome after MCAO assessed by neurological scoring according to Bederson et al.[32]. Note that significant improvement at 12 and 16 weeks of LOTUS-Tg mice is shown. (2-way repeated-measures ANOVA with Tukey multiple comparison; ***p < 0.001, ^†^*p* < 0.05, ^††^*p* < 0.01. Data are mean ± S.E.M. n = 9 per group).

Because LOTUS overexpression enhances neuronal plasticity of motor pathways, we examined whether LOTUS-Tg mice show functional recovery after ischemic stroke. As assessed by the Bederson’s neurological score, severe behavioral deficits were observed in all animals 1 week after MCAO. However, animals showed gradual improvement thereafter in LOTUS-Tg mice, and significant improvement was found at 12 weeks and 16 weeks after MCAO (Fig 5, S5 Table). Our results suggest that LOTUS overexpression promotes functional recovery in the late phase after ischemic stroke.

### RF axonal remodeling correlates with behavioral outcome after ischemic stroke

**Fig 6:**
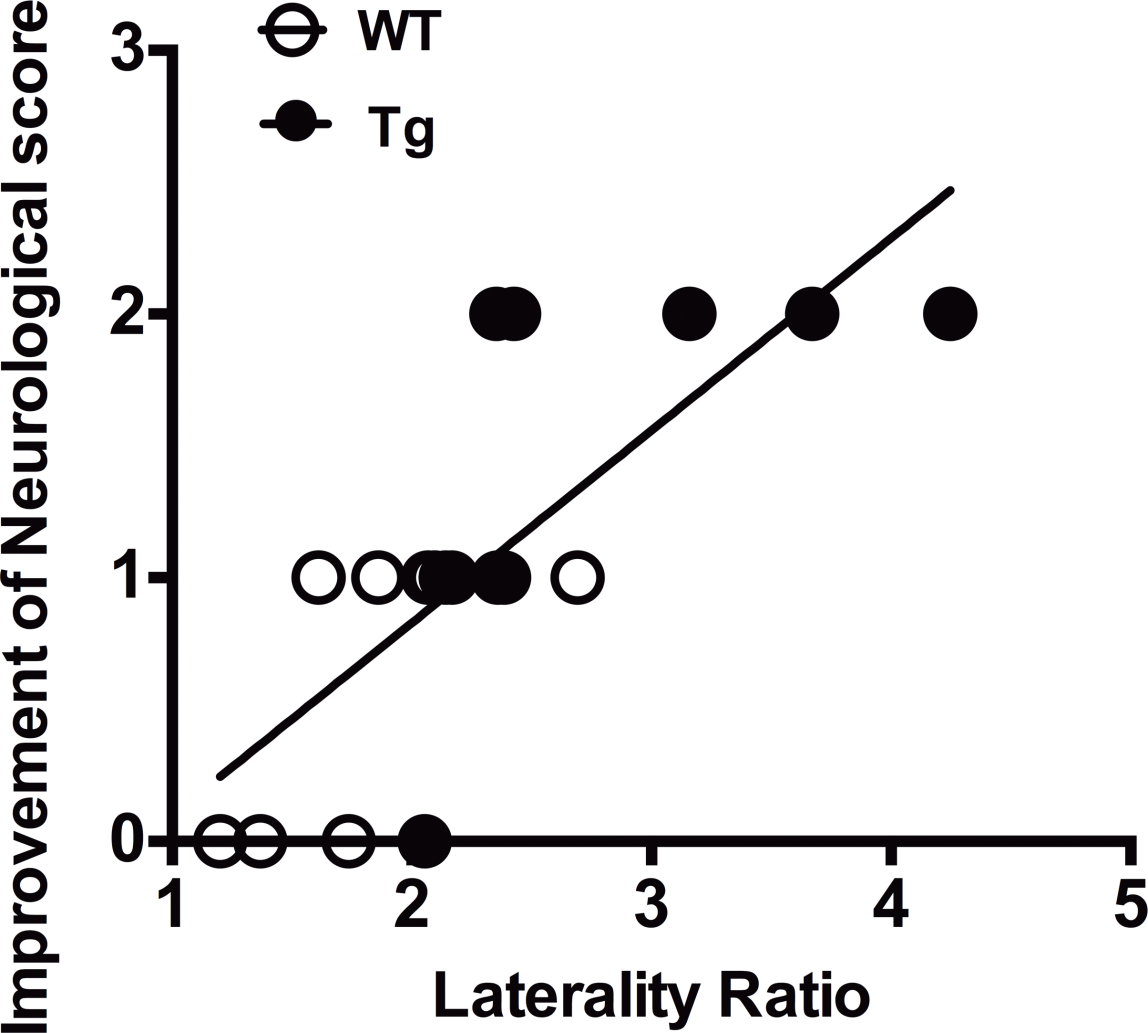
Correlation between axonal remodeling and behavioral recovery. The behavioral improvement assessed by Bederson’s neurological score was highly correlated with the laterality ratio of corticoreticular fibers of the medulla oblongata (R = 0.81, *p* < 0.001, Spearman’s Rank-Order Correlation).

To address whether motor pathway remodeling functionally associates to neurological outcome after ischemic stroke, the correlation of behavioral improvement with the laterality index of RF was analyzed. The neurological improvement was highly correlated with CST sprouting fibers to ipsi-ischemic RF (R = 0.81, *p* < 0.001, Spearman’s Rank-Order Correlation) (Fig 6).

## Discussion

**Fig 7:**
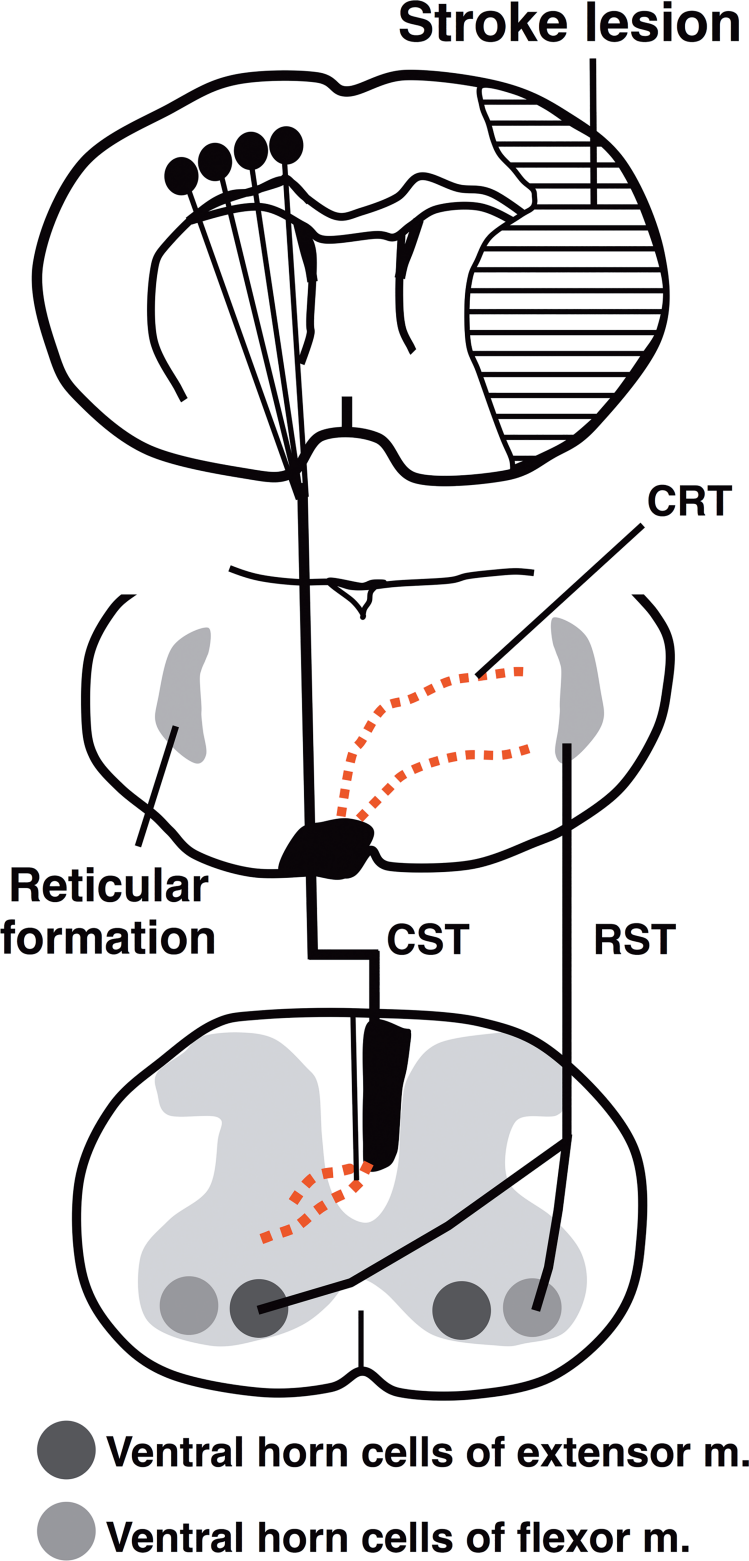
Schematic diagram showing enhancement of the midline-crossing fibers by LOTUS overexpression after MCAO. The midline-crossing corticoreticular tract (CRT) and corticospinal tract (CST) indicated by the red dashed lines from non-ischemic CST after MCAO in LOTUS-Tg mice. RST: reticulospinal tract.

Here we demonstrated that increased endogenous LOTUS expression promotes axonal remodeling of motor-associated pathways in the medulla oblongata and gray matter of the cervical spinal cord after cerebral ischemia. LOTUS overexpression improved ischemia-induced deficits on the Bederson’s neurological scale over 16 weeks after MCAO with significant correlation with an increase in CST sprouting fibers extending from the non-ischemic side to the ischemic side of RF. These findings suggest that increased LOTUS expression accelerated neuronal plasticity after ischemia with increasing axonal sprouting [23] (Fig 7).

We used older mice compared to many other stroke studies, and also used the longer occlusion time (45min) of intraluminal thread MCAO model with permanent proximal occlusion of ipsilateral ICA under strict control of body temperature in order to obtain the larger infarct volume with lower mortality and prolonged deficits (S3A and S3B Fig). On the other hand, to ensure proper occlusion of the MCA, measurement of the cerebral blood flow with LDF was performed during surgery to help minimize the standard deviation of infarct volumes [22] (S2A Fig, S3C Fig). Some measurements were unstable and not continuous in all animals tested in the cohort for chronic experiment (S2B and S2C Fig). Therefore, we first confirmed success of MCAO surgery by estimating the neurological score [19, 20], then directly and indirectly assessed the ischemic stress using two parameters of infarct volume and weight loss thereafter (Fig 1, S4 Fig and S1 Table) [17, 18].

Regarding behavioral analysis, it is challenging to quantifiably assess neurorepair or the long-term effects of neuro-protective/regenerative substances after MCAO in rodents [37–39]. Although the Bederson’s scale used in this study is somewhat unsuitable for the evaluation for long-term functional outcome, especially in animals with normal stroke volume, previous studies have reported that neurological examinations such as the Bederson score in the rodent model with large infarction did not recover quickly even in the chronic phase [18, 38, 40, 41]. Further functional assessment using rat or non-human primate would be needed for the evaluation of LOTUS effect *in vivo*.

Neuronal functions after injury are maintained by neuronal network reorganization. In the adult brain, this plastic reorganization is in part inhibited by NgR1-mediating signal transduction [9]. LOTUS blocks the interaction between NgR1 and all four types of its ligands and may hence be critical for the NgR1-signaling inhibition in the promotion of plastic reorganization, particularly under complex conditions such as ischemic stroke [15, 16]. However, the molecular dynamics of LOTUS after ischemic stroke remain uncharacterized.

Here immunoblot analysis revealed that endogenous LOTUS protein in WT mice was transiently upregulated 6 weeks after ischemia, particularly in the contralateral non-ischemic hemisphere (Fig 2B). Thus, LOTUS expression is upregulated in response to ischemia in the delayed stage through a possible compensatory mechanism in the non-ischemic hemisphere. In contrast, NgR1 and Nogo-A expression did not change throughout the observation period (Fig 2C and 2D), which is consistent with previous reports in the subcortical structures [35, 42, 43]. Furthermore, protein levels of NgR1, almost all of which is expressed in neurons [44], did not show any statistical difference at each time point in both hemispheres (S5 Fig), even though normalization of protein expression versus neuronal loss occurring in the basal ganglia cannot be performed. Taking into consideration that, given the neuronal loss occurring in the basal ganglia, LOTUS and NgR1 expression may likely be increased in the surviving neurons. There is, indeed, no direct evidence showing that overexpression of LOTUS affects NgR1 signaling in vivo. However, overexpression of LOTUS strongly suppresses NgR1 ligands-induced NgR1-signaling such as growth cone collapse and axon growth inhibition in cultured neurons [16]. Moreover, neutralization of Nogo-A and NgR1 blockades repair pathological and functional conditions of motor-related pathways, even in aged rodents and after delayed time points of experimental stroke [12, 45]. These findings suggest that NgR1-signaling is affected by overexpression of LOTUS, and that the adult brain retains the capacity to reorganize neural networks through shifting the balance of NgR1-signaling and its inhibition by LOTUS in the chronic phase of ischemic stroke.

We next asked whether NgR1-signaling suppression by increased LOTUS leads to an induction of axonal sprouting in the medulla oblongata and cervical gray matter. We could not find statistically significant increases in axonal sprouting from the contra-lateral CST in these regions of WT mice after ischemia (Fig 3B and 3C), suggesting that endogenous responses to ischemia are not sufficient to induce axonal sprouting from uninjured CST. However, in LOTUS-Tg mice, axonal sprouting of CST crossing fibers in the medulla oblongata and cervical spinal cord to the denervated side, were significantly increased compared with that in WT mice (Fig 3C, Fig 4B and 4C), indicating that LOTUS overexpression could increase the neural plasticity in these regions after ischemia. The axonal sprouting after ischemia was also accompanied by delayed motor function improvement, which correlates with the laterality ratio of sprouting axons in RF (Fig 6). Although this correlative data shows indirect relationship between LOTUS expression and functional recovery, these findings are consistent with previous reports demonstrating neuronal reconstruction in CST and the other motor-related pathways in cerebral white matter or the brainstem, such as the corpus callosum, striatum, and the corticorubral tract, by blockade of NgR1-signaling for functional recovery after brain damage including ischemic stroke [7, 10, 11, 13, 28, 46]. Therefore, the present study strongly suggests that there is a relationship between LOTUS overexpression and neuronal plasticity in brainstem.

Thus, we examined neuronal plasticity after MCAO by neuroanatomical analysis using an anterograde neuronal tracer BDA and found that ischemic stress induced axonal sprouting from the contra-ischemic CST in cervical gray matter and RF in medulla oblongata. RF is a set of interconnected nuclei located throughout the brainstem, comprising several neural networks with many functions and receives neural input from the motor cortex as CRT, as evidenced by diffusion tensor imaging in humans [47] and electrical activation by transcranial magnetic stimulation of primate motor cortex [48]. The secondary reticulospinal tract is assumed to function as an extrapyramidal system to maintain locomotor activity and posture [49, 50], which was further demonstrated by the direct activation of the ipsilateral flexor and contralateral extensor of the forelimb muscle in the primate [51]. Axonal sprouting in the gray matter of the cervical spinal cord at the C6-7 level was promoted greater than that at the C4-5 level (Fig 4B). Because the neuronal remodeling enhanced by LOTUS overexpression improved the paralysis of the extensor muscle of the denervated forelimb, overexpressed LOTUS may promote histological reconstruction both in pyramidal and extra-pyramidal tracts after MCAO. Therefore, our findings suggest that LOTUS overexpression enhanced axonal sprouting of the uninjured CST to both pyramidal and extrapyramidal systems to synergistically promote functional motor recovery after ischemic stroke.

Our data are consistent with previous studies demonstrating the importance of the blockade of NgR1-signaling for axonal sprouting from the uninjured CST and functional recovery, even during the later phase after ischemic stroke. Blockade of Nogo-A using a neutralizing antibody injected into the lateral ventricle after focal ischemia has been shown to increase midline-crossing corticorubal or corticospinal tract fibers from uninjured pyramidal neuron in rodents, accompanied by improved motor performance [7, 11]. This treatment is effective even when initiated in the chronic phase (up to 9 weeks) [10, 12]. Other reports also have demonstrated that mutant mice lacking NgR1 or treatment with Nogo inhibitor showed increased sprouting of midline-crossing coritcorubal and corticospinal tracts accompanied by motor function improvement [23, 52]. However, there is a contradictory result showing a lack of both motor function improvement and sprouting of midline-crossing fibers in Nogo-A, B mutant mice [27]. In addition, triple mutant mice (e.g., Nogo-/MAG-/OMgp-deficient mice) did not show synergistic effect on axonal regeneration after spinal cord injury [52]. Taken together, further investigation of endogenous key factors such as LOTUS would give rise to invaluable information in the regulation of NgR1-signaling for future therapeutic application to promote axonal plasticity and functional recovery after CNS damage.

Ischemic stroke is characterized by complex spatiotemporal events and heterogeneous spectrum of condition. Therefore, therapy acting on multiple targets may be preferable [36, 53, 54]. Although translation from basic research to clinical application is considered to be difficult in stroke recovery studies [55], LOTUS may have a consistent effect for neuronal plasticity and therapeutic potential for ischemic stroke [16]. Moreover, treatment strategies using endogenous proteins, such as LOTUS, would be more logical and advantageous. Further evaluation of LOTUS functional effects *in vivo* and studies using recombinant LOTUS protein in ischemic stroke would be required for potential clinical application in the future.

## Supporting information

S1 Fig Construct of LOTUS transgenic mice. Schematic representation of the construct for the generation of transgenic mice. HA-tagged mice LOTUS, comprising the mouse synapsin1 promoter, Igk-chain leader sequence, HA tag, mouse *lotus* cDNA except for the signal sequence, and Rabbit bGlobin intron/polyA (**a**). Western blotting showing LOTUS expression pattern in the CNS of P56 mice (**b, c**). (O.B.; Olfactory bulb, Cx.; Cerebral cortex, B.S.; Brainstem)
(TIFF)

S2 Fig Experimental procedures and animal groups. Mice subjected to middle cerebral artery occlusion (MCAO) were used for **(a)** infarct analysis in the acute phase **(b)** infarct analysis in the chronic phase, corticospinal tract tracing, neurological score, and **(c)** protein expression studies (immunoblots).
(TIFF)

S3 Fig No effect of LOTUS overexpression on ischemic lesion in acute phase after MCAO. **(a)** Representative coronal sections stained with TTC-reacted brain 72 h after MCAO demonstrated a representative stroke size and location in the ipsilateral sensorimotor cortex and striatum. **(b)** All infarct area (mm^2^) in each slice showed no statistical difference between groups. **(c)** CBF changes, measured by LDF, during cerebral ischemia and reperfusion are similar in WT and LOTUS-Tg groups. (2-way repeated-measures ANOVA; NS. Data are mean ± S.E.M., n = 9 in WT; n = 6 in LOTUS-Tg, Bar indicates 2 mm)
(TIFF)

S4 Fig Body weight after MCAO in WT and LOTUS-Tg group. The body weight was not statistically different between the WT and LOTUS-Tg group. (2-way repeated-measures ANOVA, F (1, 16) = 1.51, *p* = 0.24, Data are mean ± S.E.M. n = 9 per group) NS, not significant.
(TIFF)

S5 Fig NgR1 protein expression in WT and LOTUS-Tg mice after MCAO. No significant difference was seen in NgR1 expression between ipsi- and contra-ischemic hemispheres at each time point. (2-way ANOVA with post hoc analysis; Data are mean ± S.E.M. n = 3 per group) NS, not significant.
(TIFF)

S1 Table Overexpression of LOTUS does not influence brain atrophy and cortical cavitation after stroke. Ischemic infarct was clearly observed in the cortex and striatum areas of mice subjected to 45 min MCAO. No statistical difference was found in infarct areas (*p* = 0.12, 2-way repeated-measures ANOVA with Tukey post hoc analysis, n = 9), infarct volume (*p* = 0.85, unpaired *t* test, n = 9) and cortical width index (*p* = 0.64, unpaired *t* test, n = 9) between wild type mice and LOTUS-transgenic (LOTUS-Tg) mice.
(TIFF)

S2 Table The number of BDA-labeled CST fibers at the level of the medullary RF. The number of BDA-labeled CST fibers at the level of the medullary RF in LOTUS-Tg mice was not different from that in WT mice with or without ischemia (*p* = 0.68, 2-way ANOVA; non-ischemic (sham) group n = 6, ischemic group n = 9).
(TIFF)

S3 Table Percentage of uncrossed and midline-crossing fibers from CST to RF. Uncrossing fibers (CST fiber to contra-ischemic RF, 2-way ANOVA; non-ischemic (sham) group n = 6, ischemic group n = 9). In the crossing fibers (CST fiber to ipsi-ischemic RF, 2-way ANOVA; non-ischemic (sham) group n = 6, ischemic group n = 9). Laterality index (*p* = 0.047, unpaired *t* test, n = 9)
(TIFF)

S4 Table The number of midline-crossing fibers of the cervical spinal cord. The number of midline-crossing fibers of WT and LOTUS-Tg mice at the C4-5 (2-way ANOVA; non-ischemic (sham) group n = 6, ischemic group n = 9) and C6-7 (2-way ANOVA; non-ischemic (sham) group n = 6, ischemic group n = 9) levels of the cervical spinal cord.
(TIFF)

S5 Table Neurological scoring after stroke. (2-way repeated-measures ANOVA with Tukey multiple comparison, n = 9 per group).
(TIFF)
