# Supplementary figures and images for: LOTUS overexpression accelerates neuronal plasticity after focal brain ischemia in mice
Source: PLoS One. 2017 Sep 7;12(9):e0184258. doi: 10.1371/journal.pone.0184258 (PMC5589167; doi:10.1371/journal.pone.0184258)

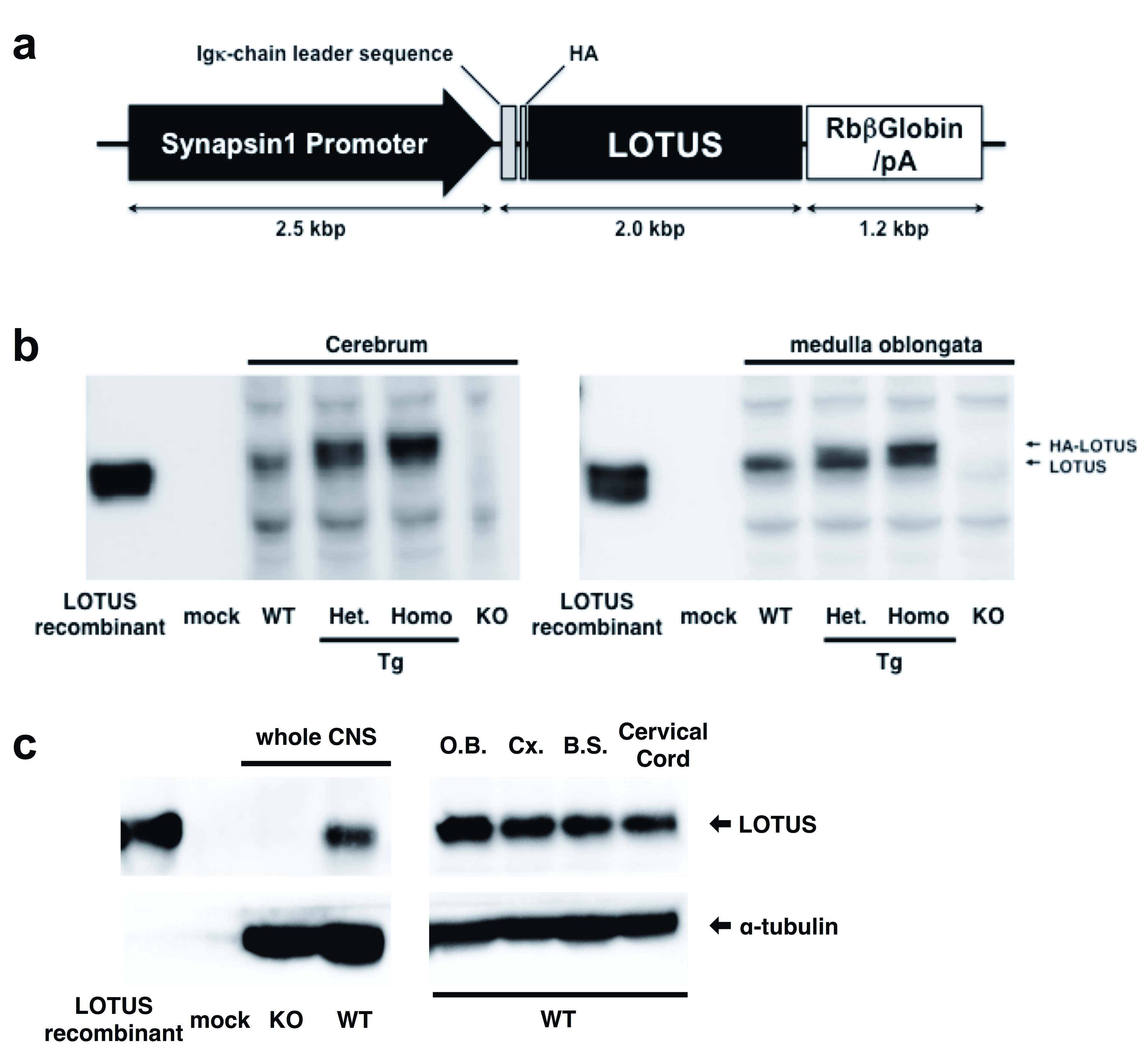

Supplement: S1 Fig — Schematic representation of the construct for the generation of transgenic mice. HA-tagged mice LOTUS, comprising the mouse synapsin1 promoter, Igk-chain leader sequence, HA tag, mouse lotus cDNA except for the signal sequence, and Rabbit bGlobin intron/polyA (a). Western blotting showing LOTUS expression pattern in the CNS of P56 mice (b, c). (O.B.; Olfactory bulb, Cx.; Cerebral cortex, B.S.; Brainstem) (TIFF) [file pone.0184258.s001.tiff]

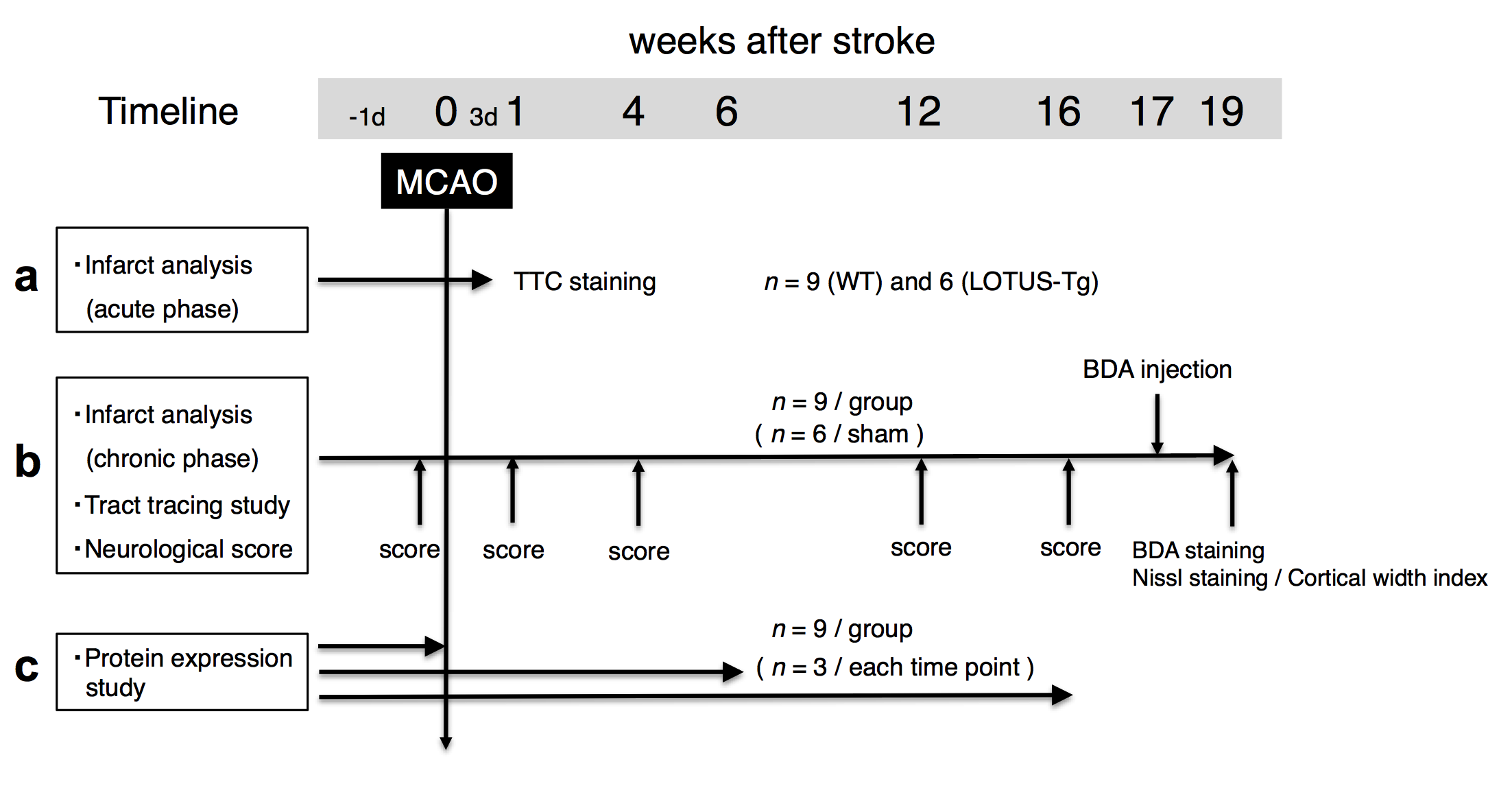

Supplement: S2 Fig — Mice subjected to middle cerebral artery occlusion (MCAO) were used for (a) infarct analysis in the acute phase (b) infarct analysis in the chronic phase, corticospinal tract tracing, neurological score, and (c) protein expression studies (immunoblots). (TIFF) [file pone.0184258.s002.tiff]

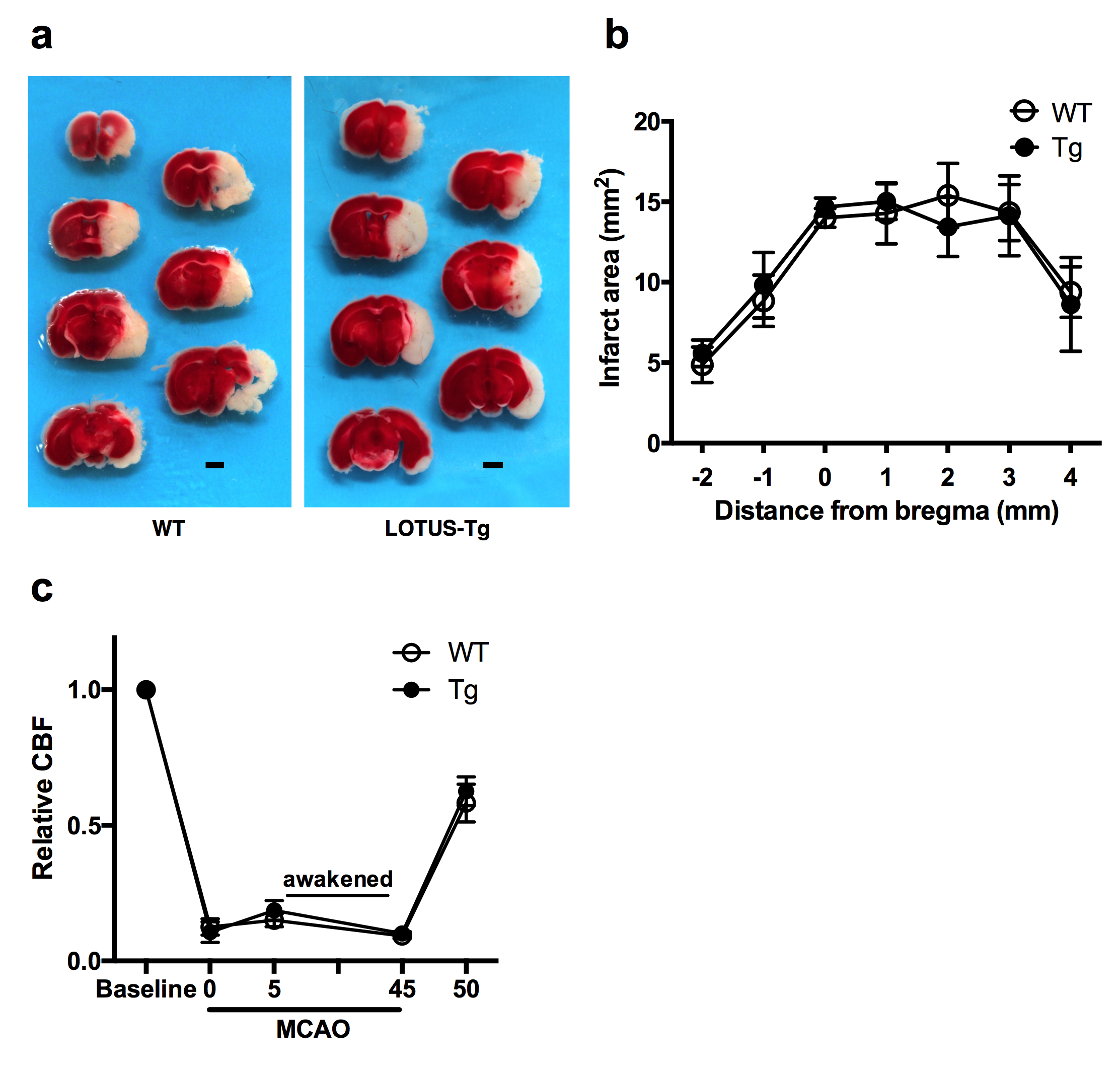

Supplement: S3 Fig — (a) Representative coronal sections stained with TTC-reacted brain 72 h after MCAO demonstrated a representative stroke size and location in the ipsilateral sensorimotor cortex and striatum. (b) All infarct area (mm2) in each slice showed no statistical difference between groups. (c) CBF changes, measured by LDF, during cerebral ischemia and reperfusion are similar in WT and LOTUS-Tg groups. (2-way repeated-measures ANOVA; NS. Data are mean ± S.E.M., n = 9 in WT; n = 6 in LOTUS-Tg, Bar indicates 2 mm) (TIFF) [file pone.0184258.s003.tiff]

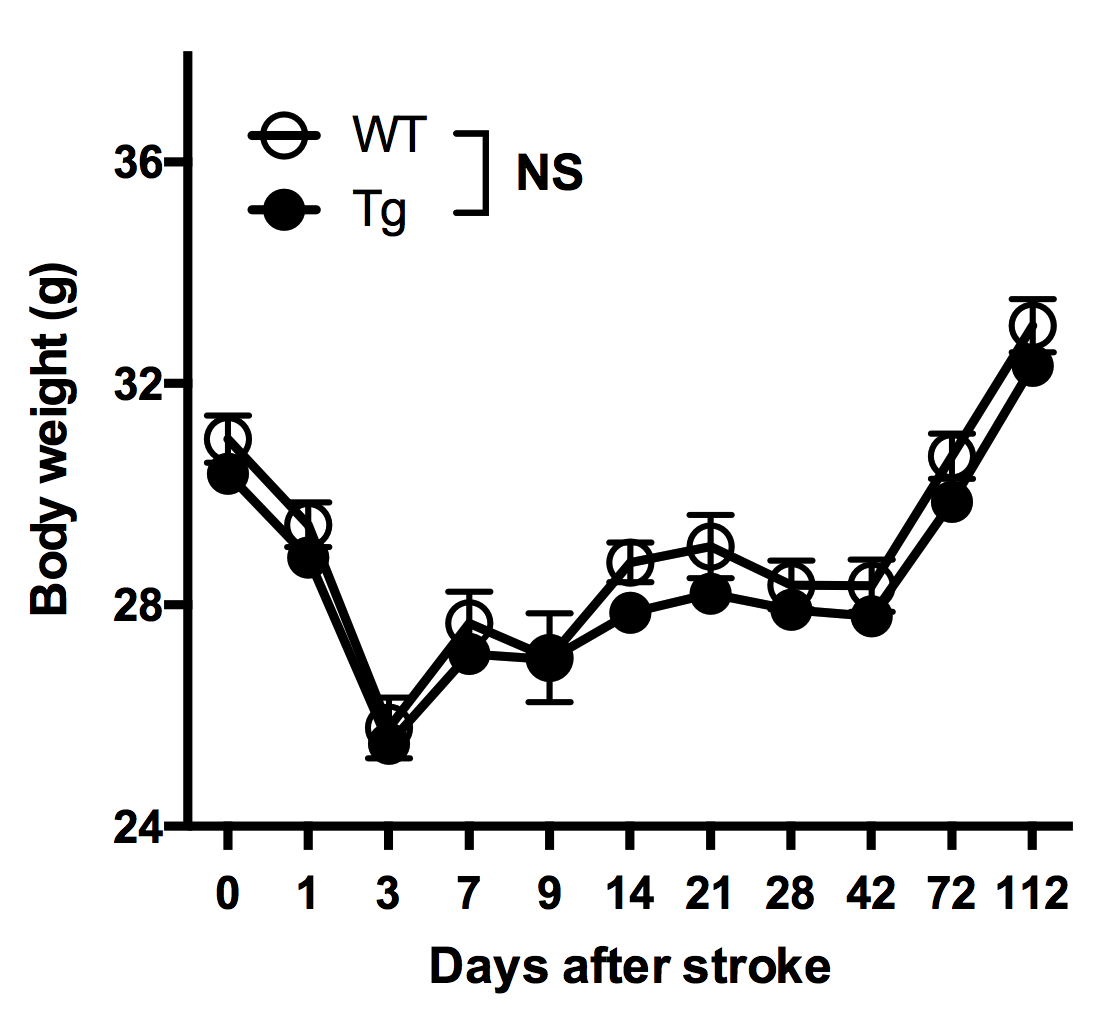

Supplement: S4 Fig — The body weight was not statistically different between the WT and LOTUS-Tg group. (2-way repeated-measures ANOVA, F (1, 16) = 1.51, p = 0.24, Data are mean ± S.E.M. n = 9 per group) NS, not significant. (TIFF) [file pone.0184258.s004.tiff]

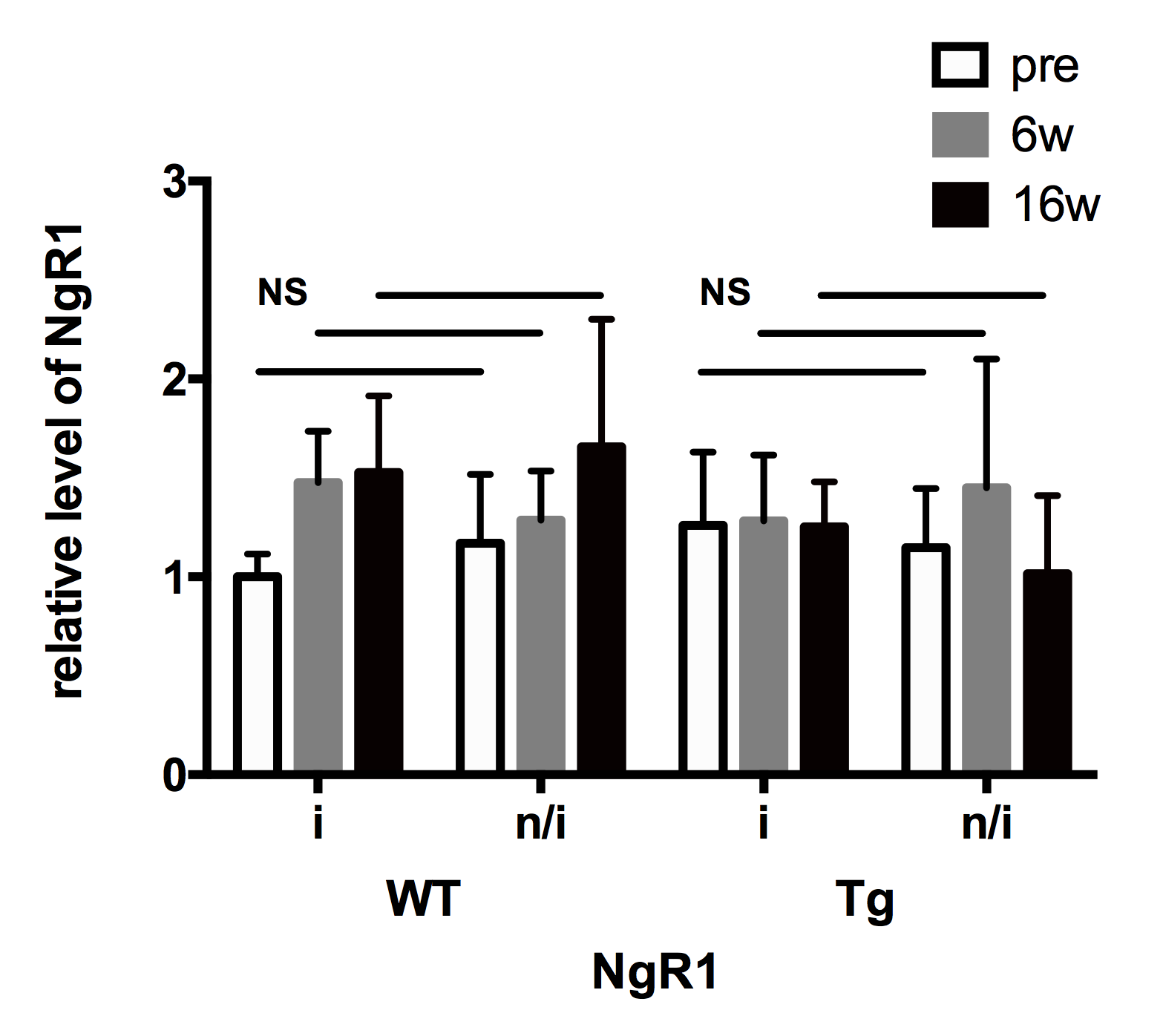

Supplement: S5 Fig — No significant difference was seen in NgR1 expression between ipsi- and contra-ischemic hemispheres at each time point. (2-way ANOVA with post hoc analysis; Data are mean ± S.E.M. n = 3 per group) NS, not significant. (TIFF) [file pone.0184258.s005.tiff]

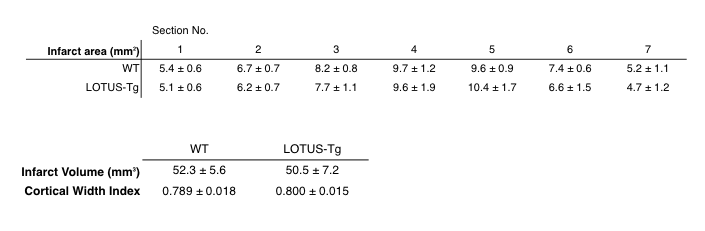

Supplement: S1 Table — Ischemic infarct was clearly observed in the cortex and striatum areas of mice subjected to 45 min MCAO. No statistical difference was found in infarct areas (p = 0.12, 2-way repeated-measures ANOVA with Tukey post hoc analysis, n = 9), infarct volume (p = 0.85, unpaired t test, n = 9) and cortical width index (p = 0.64, unpaired t test, n = 9) between wild type mice and LOTUS-transgenic (LOTUS-Tg) mice. (TIFF) [file pone.0184258.s006.tiff]

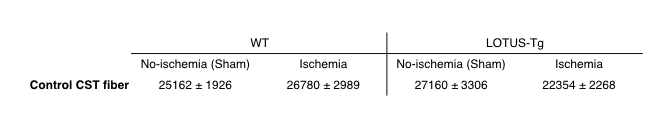

Supplement: S2 Table — The number of BDA-labeled CST fibers at the level of the medullary RF in LOTUS-Tg mice was not different from that in WT mice with or without ischemia (p = 0.68, 2-way ANOVA; non-ischemic (sham) group n = 6, ischemic group n = 9). (TIFF) [file pone.0184258.s007.tiff]

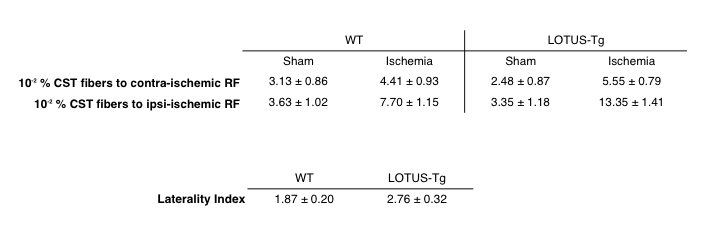

Supplement: S3 Table — Uncrossing fibers (CST fiber to contra-ischemic RF, 2-way ANOVA; non-ischemic (sham) group n = 6, ischemic group n = 9). In the crossing fibers (CST fiber to ipsi-ischemic RF, 2-way ANOVA; non-ischemic (sham) group n = 6, ischemic group n = 9). Laterality index (p = 0.047, unpaired t test, n = 9) (TIFF) [file pone.0184258.s008.tiff]

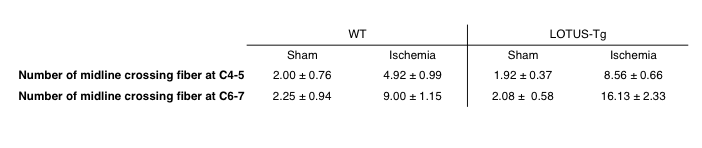

Supplement: S4 Table — The number of midline-crossing fibers of WT and LOTUS-Tg mice at the C4-5 (2-way ANOVA; non-ischemic (sham) group n = 6, ischemic group n = 9) and C6-7 (2-way ANOVA; non-ischemic (sham) group n = 6, ischemic group n = 9) levels of the cervical spinal cord. (TIFF) [file pone.0184258.s009.tiff]

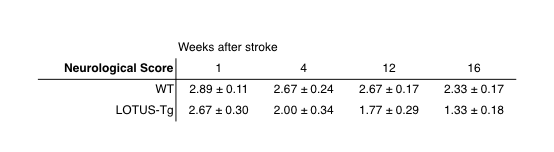

Supplement: S5 Table — (2-way repeated-measures ANOVA with Tukey multiple comparison, n = 9 per group). (TIFF) [file pone.0184258.s010.tiff]
